# Direct One-Pot Synthesis of Primary 4-Amino-2,3-diaryl- quinolines *via* Suzuki-Miyaura Cross-Coupling of 2-Aryl-4-azido-3-iodoquinolines with Arylboronic Acids

**DOI:** 10.3390/molecules16118958

**Published:** 2011-10-25

**Authors:** Malose Jack Mphahlele, Mamasegare Mabel Mphahlele

**Affiliations:** Department of Chemistry, College of Science, Engineering and Technology, University of South Africa, P.O. Box 392, Pretoria 0003, South Africa

**Keywords:** 2-aryl-4-azido-3-iodoquinolines, Suzuki-Miyaura cross-coupling, symmetrical biaryls, 4-azido-2,3-diarylquinolines, 4-amino-2,3-diarylquinolines

## Abstract

Palladium-catalyzed Suzuki-Miyaura cross-coupling of 2-aryl-4-azido-3-iodo-quinolines with arylboronic acids afforded the corresponding primary 4-amino-2,3-diarylquinolines in a single-pot operation along with symmetrical biaryls and traces of the 2,3-diaryl-4-azidoquinolines. A plausible mechanism, which implicates palladium hydride species in the reduction of the incipient 2,3-diaryl-4-azidoquinolines to afford the 4-amino-2,3-diarylquinolines is proposed.

## 1. Introduction

Our continued interest in the synthesis of primary 4-aminoquinoline derivatives stems from their importance as antimalarial, anti-inflammatory, antibacterial, and antihypertensive agents [1,2,3,4] as well as immunostimulants and non-nucleoside HIV-1 inhibitors [5,6]. Aryl substituted quinoline derivatives are also known to serve as potent inhibitors of tyrosine kinase PDGF-RTK [7]. Moreover, the 4-amino-2-arylquinolines have also been found to represent a novel class of NR1/2B subtype selective *N*-methyl-D-aspartate (NMDA) receptor antagonists [8]. Although there are several methods described in the literature for the synthesis of primary 4-amino-2-arylquinolines [6,9,10,11,12,13], corresponding data for the synthesis of 2,3-disubstituted 4-aminoquinoline derivatives is considerably less well documented [13,14]. These polysubstituted quinoline derivatives are not accessible *via* classical methods such as the Skraup, Doebner-von Miller, Friedlander and Combes syntheses [15]. Consequently, an indirect approach to efficiently functionalize the presynthesized halogenated quinoline derivatives via nucleophilic displacement and/or metal-catalyzed cross-coupling reactions leading to C*sp*^2^–N and/or C*sp*^2^–C*sp*^2^ bond formation remains the method of choice. The 4-(2-methylphenylamino)-3-iodo-quinolines, for example, were previously subjected to palladium-catalyzed Heck reaction with terminal alkenes to afford the corresponding 3-vinylquinolines with gastric H^+^/K^+^-ATPase inhibitory activity [16]. On the other hand, the analogous 2-aryl-3-iodo-4-(phenylamino)quinolines were found to undergo one-pot palladium mediated C–I and C–H bond activation and subsequent Suzuki-Miyaura cross-coupling with arylboronic acids to afford mixtures of the 2,3-diaryl-4-(phenylamino)quinolines and 2-aryl-4-([(1,1'-biaryl)-2-yl]amino)quinoline derivatives [17]. Hitherto this investigation, the Staudinger reaction of 2-aryl-4-azido-3-halogenoquinolines (X = Br, I) with triphenylphosphine in refluxing tetrahydrofuran afforded the corresponding 2-aryl-3-halogeno-4-(triphenylphosphoran-ylideneamino)quinolines [18]. The latter were either hydrolyzed to the corresponding 4-amino-2-aryl-3-halogenoquinolines or subjected to palladium-catalyzed Suzuki-Miyaura cross-coupling with phenylboronic acid followed by acetic acid-promoted hydrolysis of the incipient 2,3-diaryl-phosphazenes to afford the 4-amino-2,3-diarylquinolines [18].

A literature search revealed two papers describing the outcome of tetrakis(triphenylphosphine)-palladium(0) [Pd(PPh_3_)_4_]-catalyzed Suzuki-Miyaura cross-couplings of 4-azido-3-bromopyridine with heteroarylboronic acids [19] and 1-azido-2-bromobenzene with a series of aryl- and heteroarylboronic acids [20]. In one case, involving the coupling of 1-azido-2-bromobenzene with 2-thiopheneboronic acid, the authors isolated 2-(2-azidophenyl)thiophene (40%), bromoaniline (2%) and 2-thiophenylaniline (3%) as products [20]. They attributed the formation of bromoaniline and 2-thiophenylaniline to hydrolysis of the corresponding incipient iminophosphorane resulting from the reaction between triphenylphosphine ligand and the azide function [20]. Prompted by this literature observation and the ability of iodine to facilitate metal-catalyzed carbon-carbon bond formation of the 2-aryl-3-iodo-4-(triphenylphosphoranylideneamino)quinolines [18] we decided to investigate the reactivity of the known 2-aryl-4-azido-3-iodoquinolines in palladium-catalyzed Suzuki-Miyaura cross-couplings. The main aim of this investigation was to assess the possibility of effecting direct one-pot synthesis of the primary 4-amino-2,3-diarylquinolines via Suzuki-Miyaura cross-coupling of the 2-aryl-4-azido-3-iodoquinolines with arylboronic acids.

## 2. Results and Discussion

The Suzuki-Miyaura reaction of aryl or heteroaryl halides with arylboronic acids is a well established procedure and its mechanism generally involves: (i) oxidative addition of an aryl halide to a Pd(0) active catalyst; (ii) transmetallation of Ar–Pd–X with Ar'B(OH)_3_^−^M^+^; and (iii) reductive elimination to give a biaryl product [21,22]. The efficiency of a palladium catalyst, on the other hand, depends strongly on the ligand of palladium atom and the overall reactivity also depends on the nature of the palladium(0) complex precursor [23,24]. With this consideration in mind, we reacted the 4-azido-2-aryl-3-iodoquinolines **1a**,**b** with phenylboronic acid (1.2 equiv.) in refluxing DMF in the presence of Pd(PPh_3_)_4_ and 2 M K_2_CO_3_ as a base as a reference starting point for exploration based on literature precedents. After 48 h we isolated by column chromatography on silica gel three products in sequence, which were identified using a combination of spectroscopic techniques as the symmetrical biphenyl **2a** (3%, 5%) 2,3-diaryl-4-azidoquinolines **3a** (6%), **b** (7%) and 4-amino-2,3-diarylquinolines **4a** (15%), **b** (35%), respectively. Under similar reaction conditions using palladium acetate as pre-catalyst, phenylboronic acid (1.5–2 equiv.) and 2 M K_2_CO_3_ as a base and DMF as solvent, we also isolated by column chromatography on silica gel after 48 h the biphenyl **2a** (21%, 26%), 4-azido-2,3-diarylquinolines **3c** (14%), **d** (14%) and 4-amino-2,3-diarylquinolines **4c** (30%), **d** (50%) in sequence. Analytical data for products **4** was found to compare favourably with those of the corresponding derivatives prepared as described in our previous communication [18]. The ^1^H-NMR and ^13^C-NMR spectra of the azido derivatives **3**, on the other hand, revealed the presence of an increased number of signals in the aromatic region, which distinguished these products from the corresponding precursors. The presence of strong IR absorption band in the υ_max_ 2,110–2,119 cm^−1^ (asymmetric) and υ_max_ 1,220–1,260 cm^−1^ (symmetric) regions further distinguished systems **3** from the amino derivatives **4**. The analogous 4-phenyl-5-azidoquinolines on the other hand, previously afforded in refluxing xylene pyrido[2,3,4-*kl*]acridines via an intramolecular nitrene insertion reaction [25]. The 3-aryl-4-azido-7-methoxyquinolin-2(1*H*)-ones prepared from the reaction of 3-aryl-4-(chloro/tosyloxy)-7-methoxy-quinolin-2(1*H*)-ones with sodium azide in refluxing DMF were also found to undergo thermolytic ring closure to afford the 5-alkyl-3-methoxy-11*H*-indolo[3,2-*c*]quinolin-6(5*H*)-ones [26]. In the current investigation, no products resulting from thermolytic ring closure of **3** were isolated from the reaction mixtures.

**Scheme 1:**
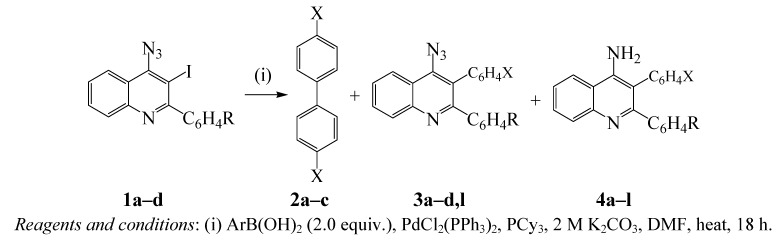
PdCl_2_(PPh_3_)_2_-PCy_3_ catalyzed Suzuki-Miyaura cross-coupling of **1** with ArB(OH)_2_.

Despite the outcome of the above reaction, we were concerned about the low yields and prolonged reaction times, presumably due to the slow oxidative addition step using Pd(PPh_3_)_4_ as precursor of the palladium(0) complex. This slow oxidative addition step is attributed to the inhibiting role of the extra PPh_3_ generated in the second equilibrium {*S*Pd(0)(PPh_3_)_3_
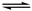
*S*Pd(0)(PPh_3_)_2_ + PPh_3_ (*K*_2_/[PPh_3_] << 1); *S* = solvent} to afford the low reactivity ligated 14-electron species (Pd(0)(PPh_3_)_2_) [24]. Conversely, the oxidative addition performed by the palladium(0) complex (Pd(0)(PPh_3_)_2_Cl^−^) generated by the reduction of dichlorobis(triphenylphosphine)palladium(II) (PdCl_2_(PPh_3_)_2_) is reported to be more than 30 times faster than that performed from Pd(0)(PPh_3_)_4_ [24]. Likewise, alkylphosphine ligands are known to coordinate with palladium and increase its electron density more than arylphosphines and, in turn, accelerate the oxidative addition and reductive elimination steps in the catalytic cycle [27,28]. Consequently, we subjected substrates **1a–d** to 2 equiv. of phenylboronic acid in the presence of PdCl_2_(PPh_3_)_2_-tricyclohexylphosphine (PCy_3_) catalyst mixture and 2 M potassium carbonate in DMF under reflux (Scheme 1). The reaction in the presence of PdCl_2_(PPh_3_)_2_-PCy_3_ catalyst mixture was complete within 18 h. Analysis of the crude product mixtures by thin layer chromatography revealed in all cases three spots of different polarity and intensity with no traces of the spot corresponding to the starting material. The mixture was isolated by column chromatography on silica gel to afford the biphenyl **2a**, 4-azido-2,3-diarylquinolines **3a–d** (minor) and 4-amino-2,3-diarylquinolines **4a–d** (major) in sequence. The reaction conditions were also extended to include 4-fluorophenylboronic and 4-methoxyphenylboronic acids as coupling partners. Although in all cases, traces of the 4-azido-2,3-diarylquinolines **3** (2nd spot) were detected by thin layer chromatography in the crude product mixture, careful column chromatographic separation on silica gel in most cases led to isolation of the self-coupled biaryl derivatives **2b,c** (minor) and the 4-amino-2,3-diarylquinolines **4a–l** as the major products.

**Table:** 

**3/4**	**4-R**	**4-X**	**% Yield 2**	**% Yield 3**	**% Yield 4**
**a**	4-H	4-H	24 ( **2a**)	12	57
**b**	4-F	4-H	24 ( **2a**)	16	65
**c**	4-Cl	4-H	24 ( **2a**)	10	54
**d**	4-OMe	4-H	24 ( **2a**)	11	66
**e**	4-H	4-F	20 ( **2b**)	-	65
**f**	4-F	4-F	20 ( **2b**)	-	66
**g**	4-Cl	4-F	20 ( **2b**)	-	56
**h**	4-OMe	4-F	20 ( **2b**)	-	64
**i**	4-H	4-MeO	17 ( **2c**)	-	63
**j**	4-F	4-MeO	17 ( **2c**)		57
**k**	4-Cl	4-MeO	17 ( **2c**)	-	60
**l**	4-OMe	4-MeO	17 ( **2c**)	9	68

At first glance, we also thought products **4** are the result of the initial cross-coupling of **1** with arylboronic acids and subsequent *in situ *Staudinger reaction of the 2,3-diaryl-4-azidoquinolines **3** with PPh_3_ released from the catalyst followed by hydrolysis of the incipient 2,3-diaryl-4-(triphenyl-phosphoranylideneamino)quinolones, in analogy with the previous literature observation [20]. However, this possibility was ruled out by the absence of triphenylphosphonium oxide in the reaction mixture or crude product (tlc monitoring or ^31^P-NMR), which is the expected by-product of hydrolysis of phosphazene derivatives [18]. Recourse to literature, revealed a paper describing the results of palladium acetate (Pd(OAc)_2_)-catalyzed Suzuki-Miyaura cross-coupling of nitroaryl halides with arylboronic acids in DMF/H_2_O at 150 °C using K_2_CO_3_ as a base in the absence or presence of a ligand (PPh_3_ or DABCO) [29]. The reaction afforded the corresponding biaryl derivatives with simultaneous reduction of nitro- to amino group and the authors attributed the reduction of the nitro group to molecular hydrogen based on literature precedent [30]. However, DMF-water mixture failed to reduce nitrobenzene to aniline at 150 °C [29]. Moreover, PdCl_2_(PPh_3_)_2_-PCy_3_ catalyzed cross-coupling of **1d** with PhB(OH)_2_ using 2 M K_2_CO_3_ in dioxane also afforded **2a** (26%), **3d** (11%) and **4d** (62%) in sequence. We envisioned that molecular hydrogen generated from DMF-water medium in the presence of Pd(PPh_3_)_4_ or PdCl_2_(PPh_3_)_2_ would also hydrogenolyze the azidoiodoquinolines **1** in analogy with literature observation for the selective hydrogenolysis of azidoiodoarenes by H_2_-Pd/C mixture to afford the azidoarenes [31].

**Scheme 2:**
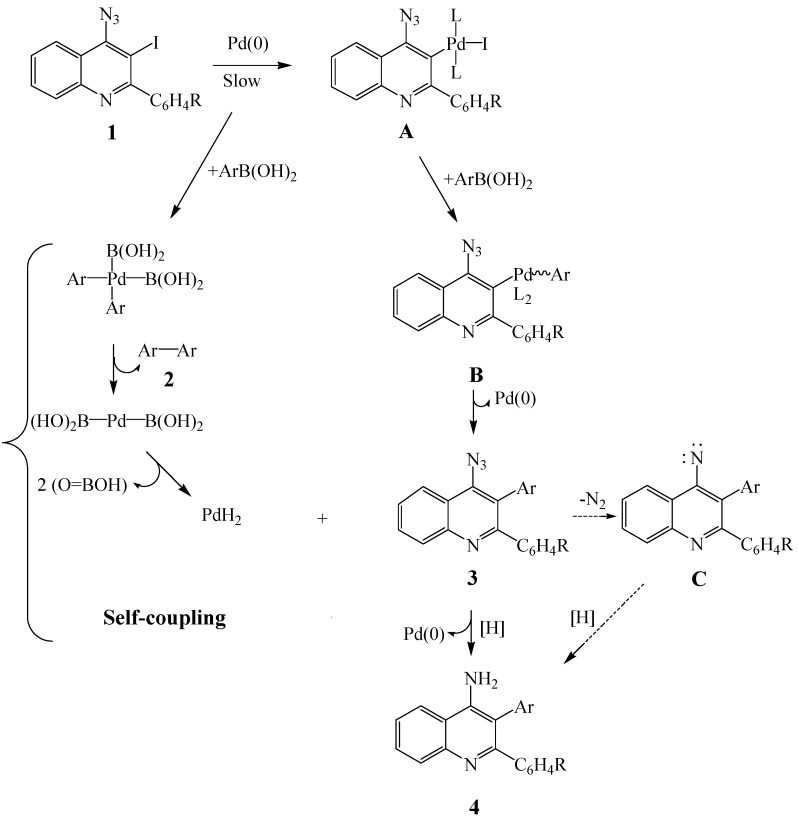
Proposed mechanism for the one-pot Suzuki-Miyaura cross-coupling and reduction of **1** incorporating self-coupling of ArB(OH)_2_.

The intriguing results observed in this investigation prompted us to propose a mechanism outlined in Scheme 2 to account for the one-pot palladium-catalyzed cross-coupling and subsequent reduction of the azido group to afford the primary 4-aminoquinolines **4**. The symmetrical biaryls **2** are the result of the self-coupling of aryl groups from arylboronic acid. Homo-coupling of arylboronic acids is a side reaction usually observed for the Suzuki-Miyaura cross-coupling reactions under both Pd(PPh_3_)_4_ and Pd(OAc)_2_ catalysis especially when the cross-coupling is very slow [32,33]. The self-coupling step is known to be accompanied by the release of palladium hydride (PdH_2_) along with metaboric acid (HOB=O) liberated in the form of borate under alkaline aqueous medium used in the Suzuki-Miyaura cross-coupling reactions [32]. The intermediate palladium hydride released during the catalytic cycle may either release hydrogen or serve as hydride source to reduce oxidants present in the reaction media and generate Pd(0) [32]. Although palladium hydride is implicated in the self-coupling mechanism [32], palladium hydrides L_2_PdHCl [L = PCy_3_ or P(*t*-Bu)_3_], have been observed during the course of palladium-catalyzed Heck reaction [34].

The envisioned self-coupling step is presumably accompanied by a slow oxidative addition of palladium(0) complex into **1** to form **A**, followed by transmetallation and reductive elimination from **B** to afford the corresponding 2,3-diaryl-4-azidoquinoline **3** (detected by tlc or isolated by column chromatography) as invoked in the classical Suzuki-Miyaura cross-coupling reaction mechanism. We envision that intermediate **3** is reduced by palladium hydride (PdH_2_ or L_2_PdHI) released from the self-coupling reaction to afford the 4-amino-2,3-diarylquinoline **4** in moderate yields. The possibility of formation of the latter via reduction of a nitrene intermediate **C** generated from **3** cannot be completely ruled out. Despite the fact that our proposed mechanism is necessarily speculative, it represents the best option consistent with the formation of the observed products in the presence or absence of PCy_3_.

## 3. Experimental

### 3.1. General

Melting points were recorded on a Thermocouple digital melting point apparatus and are uncorrected. IR spectra were recorded as powders using a FTS 7000 Series Digilab Win-IR Pro ATR (attenuated total reflectance) spectrometer. For column chromatography, Merck kieselgel 60 (0.063–0.200 mm) was used as stationary phase. NMR spectra were obtained as CDCl_3_ solutions using Varian Mercury 300 MHz NMR spectrometer and the chemical shifts are quoted relative to the solvent peaks. Low- and high-resolution mass spectra were recorded at an ionization potential of 70 eV using Micromass Autospec-TOF (double focusing high resolution) instrument. The synthesis and characterization of substrates **1** have been described elsewhere [18].

### 3.2. Typical Procedure for the PdCl_2_(PPh_3_)_2_-PCy_3_ Catalyzed Cross-Coupling Reactions of ***1*** with ArB(OH)_2_

#### 3.2.1. Biphenyl (**2a**), 4-Azido-2,3-diphenylquinoline (**3a**) and 4-Amino-2,3-diphenylquinoline (**4a**)

A mixture of **1a** (0.20 g, 0.54 mmol), phenylboronic acid (0.13 g, 1.08 mmol), 2M K_2_CO_3_ (1.2 mL), PdCl_2_(PPh_3_)_2_ (0.02 g, 0.03 mmol) and PCy_3_ (0.02 g, 0.05 mmol) in DMF (5 mL) in a two-necked flask equipped with a stirrer bar, rubber septum and a condenser was flushed for 30 min with argon gas. A balloon filled with argon gas was connected to the top of the condenser and the mixture was heated with stirring at 80–90 °C under argon atmosphere for 18 h and then allowed to cool to room temperature. The cooled mixture was poured into a ice-cold water and the product was taken-up into chloroform. The combined organic extracts were washed with brine, dried over anhydrous MgSO_4_, filtered and then evaporated under reduced pressure. The residue was purified by column chromatography on silica gel (20% ethyl acetate-hexane) to afford **2a**, **3a** and **4a** in sequence.

*Biphenyl* (**2a**). Solid (19.5 mg, 24%), mp 69–71 °C (ethanol) (Lit. [35] 70–72 °C), *R*_F_ 0.94.

*4-Azido-2,3-diphenylquinoline* (**3a**). Solid (20 mg, 12%) mp 125–127 °C, *R*_F_ 0.63; ν_max_ (neat) 764, 838, 1174, 1240, 1379, 2110 cm^−1^; δ_H_ (300 MHz, CDCl_3_) 7.19–7.22 (m, 3H), 7.25–7.35 (m, 6H), 7.55–7.60 (m, 2H), 7.76 (dt, *J* 1.5 and 7.8 Hz, 1H), 8.15 (dd, *J* 0.6 and 8.7 Hz, 1H), 8.23 (d, *J* 8.7 Hz, 1H); δ_C_ (75 MHz, CDCl_3_) 123.0, 126.5, 126.8, 127.7, 127.8, 128.3, 128.4, 129.0, 129.4, 129.6, 130.3, 131.5, 134.5, 140.1, 142.4, 147.8, 159.6; *m/z *295 (100, MH^+^-N_2_), HRMS (ES): MH^+^-N_2_ found 295.1237. C_21_H_15_N_2_^+^ requires 295.1235.

*4-Amino-2,3-diphenylquinoline* (**4a**). Solid (90 mg, 57%), mp 238–240 °C (Lit. [18] 239–241 °C), *R*_F_ 0.15.

#### 3.2.2. Biphenyl (**2a**), 4-Azido-2-(4-fluorophenyl)-3-phenylquinoline (**3b**) and 4-Amino-2-(4-fluoro-phenyl)-3-phenylquinoline (**4b**)

A mixture of **1b** (0.50 g, 1.28 mmol), phenylboronic acid (0.31 g, 2.56 mmol), PdCl_2_(PPh_3_)_2_ (0.04 g, 0.06 mmol), PCy_3_ (0.04 g, 0.13 mmol), 2 M K_2_CO_3_ (2.6 mL) in DMF (10 mL) was treated as described above. Work-up and column chromatography on silica gel (20% ethyl acetate-hexane) afforded **2a** (19.6 mg, 10%), *R*_F_ 0.90; **3b** and **4b** in sequence.

*4-Azido-2-(4-fluorophenyl)-3-phenylquinoline* (**3b**). Solid (69.6 mg, 16%) mp 131–133 °C, *R*_F_ 0.63; ν_max_ (neat) 764, 838, 1159, 1221, 1364, 1480, 2110 cm^−1^; ^1^H-NMR δ_H_ (300 MHz, CDCl_3_) 6.90 (t, *J* 8.7 Hz, 2H), 7.24–7.30 (m, 4H), 7.35–7.38 (m, 3H), 7.59 (dt, *J* 1.2 and 7.5 Hz, 1H), 7.77 (dt, *J* 1.5 and 7.8 Hz, 1H), 8.13 (d, *J* 8.4 Hz, 1H), 8.23 (dd, *J* 1.2 and 8.4 Hz, 1H); ^13^C-NMR δ_C_ (75 MHz, CDCl_3_) 114.7 (d, ^2^*J*_CF_ 21.6 Hz), 121.5, 123.0, 126.3, 126.9, 128.5, 128.6, 129.4, 130.4, 131.4, 131.5 (d, ^3^*J*_CF_ 8.3 Hz), 134.4, 136.2 (d, ^4^*J*_CF_ 3.6 Hz), 142.6, 147.8, 158.5, 162.4 (d, ^1^*J*_CF_ 246.2 Hz); *m/z *313 (100, MH^+^-N_2_), HRMS (ES): MH^+^-N_2_ found 313.1146. C_21_H_14_FN_2_^+^ requires 313.1141.

*4-Amino-2-(4-fluorophenyl)-3-phenylquinoline* (**4b**). Solid (260 mg, 65%), mp 225–228 °C (Lit. [18] 215–217 °C), *R*_F_ 0.23.

#### 3.2.3. Biphenyl (**2a**), 4-Azido-2-(4-chlorophenyl)-3-phenylquinoline (**3c**) and 4-Amino-2-(4-chloro-phenyl)-3-phenylquinoline (**4c**)

A mixture of **1c** (0.25 g, 0.62 mmol), phenylboronic acid (0.15 g, 1.23 mmol), PdCl_2_(PPh_3_)_2_ (0.02 g, 0.03 mmol), PCy_3_ (0.20 g, 0.06 mmol), 2 M K_2_CO_3_ (1.2 mL) in DMF (6 mL) was treated as described above. Work-up and column chromatography on silica gel (20% ethyl acetate-hexane) afforded **2a** (20.2 mg, 21%), *R*_F_ 0.90, **3c** and **4c** in sequence.

*4-Azido-2-(4-chlorophenyl)-3-phenylquinoline* (**3c**). Solid (19 mg, 10%), mp 140–141 °C (ethanol), *R*_F_ (20% ethyl acetate-hexane) 0.54; ν_max_ (neat) 838, 1159, 1221, 1364, 1504, 2110 cm^−1^; ^1^H-NMR δ_H_ (300 MHz, CDCl_3_) 7.16–7.20 (m, 2H), 7.22–7.28 (m, 4H), 7.33–7.38 (m, 3H), 7.58 (dt, *J* 1.2 and 8.1 Hz, 1H), 7.76 (dt, *J* 1.5 and 7.8 Hz, 1H), 8.12 (dd, *J* 1.5 and 8.4 Hz, 1H), 8.22 (dd, *J* 1.5 and 8.4 Hz, 1H); ^13^C-NMR δ_C_ (75 MHz, CDCl_3_) 121.5, 123.0, 126.2, 127.0, 127.9, 128.5, 128.6, 129.4, 130.4, 131.0, 131.5, 134.0, 134.2, 138.6, 142.6, 147.8, 158.2; *m/z *329 (100, MH^+^-N_2_), HRMS (ES): MH^+^-N_2_ found 329.0846. C_21_H_14_N_2_^35^Cl^+^ requires 329.0845.

*4-Amino-2-(4-chlorophenyl)-3-phenylquinoline* (**4c**). Solid (130.7 mg, 64%), mp 228–231 °C (ethanol) (Lit. [18] 237–239 °C), *R*_F_ 0.09.

3.2.4. Biphenyl (**2a**), 4-Azido-2-(4-methoxyphenyl)-3-phenylquinoline (**3d**) and 4-Amino-2-(4-methoxyphenyl)-3-phenylquinoline (**4d**)

A mixture of **1d** (0.30 g, 0.76 mmol), phenylboronic acid (0.18 g, 1.49 mmol), PdCl_2_(PPh_3_)_2_ (0.03 g, 0.04 mmol), PCy_3_ (0.02 g, 0.07 mmol), 2 M K_2_CO_3_ (1.5 mL) in DMF (6 mL) was treated as described above. Work-up and column chromatography on silica gel (20% ethyl acetate-hexane) afforded **2a** (0.02 g, 17%), *R*_F_ 0.87, **3d** and **4d** in sequence.

*4-Azido-2-(4-methoxyphenyl)-3-phenylquinoline* (**3d**). Solid (27.8 mg, 16%), mp 135–136 °C (ethanol), *R*_F_ 0.56; ν_max_ (neat) 670, 743, 1030, 1247, 1504, 2110 cm^−1^; ^1^H-NMR δ_H_ (300 MHz, CDCl_3_) 3.76 (s, 3H), 6.73 (d, *J* 8.7 Hz, 2H), 7.19–7.22 (m, 2H), 7.27–7.35 (m, 5H), 7.64 (dt, *J* 1.5 and 8.1 Hz, 1H), 7.78 (dt, *J* 1.5 and 8.7 Hz, 1H), 8.19 (dd, *J* 0.9 and 8.1 Hz, 1H), 8.30 (dd, *J* 1.2 and 7.8 Hz, 1H); ^13^C-NMR δ_C_ (75 MHz, CDCl_3_) 55.2, 113.2, 124.6, 125.3, 127.4, 127.6, 128.1, 129.7, 130.2, 130.7, 131.3, 132.7, 132.8, 137.4, 141.8, 147.7, 158.7, 159.4; *m/z *353 (30, MH^+^), 325.1346 (100, MH^+^-N_2_), HRMS (ES): MH^+^ found 353.1414. C_22_H_17_N_4_O^+^ requires 353.1402.

*4-Amino-2-(4-methoxyphenyl)-3-phenylquinoline* (**4d**). Solid (160 mg, 66%), mp 166–169 °C (ethanol) (Lit. [18] 165–167 °C), *R*_F_ 0.10.

#### 3.2.5. 4,4'-Difluoro-1,1'-biphenyl (**2b**) and 4-Amino-3-(4-fluorophenyl)-2-(phenyl)quinoline (**4e**)

A mixture of **1a** (0.20 g, 0.54 mmol), 4-fluorophenylboronic acid (0.15 g, 1.08 mmol), PdCl_2_(PPh_3_)_2_ (0.02 g, 0.03 mmol), PCy_3_ (0.02 g, 0.05 mmol), 2 M K_2_CO_3_ (1.2 mL) in DMF (6 mL) was treated as described above. Work-up and column chromatography on silica gel (20% ethyl acetate-hexane) afforded **2b** and **4e** in sequence.

*1-Fluoro-4-(4-fluorophenyl)benzene* (**2b**). Solid (19.8 mg, 20%), mp 85–87 °C (ethanol) (Lit. [35] 87–88 °C), *R*_F_ 0.94.

*4-Amino-3-(4-fluorophenyl)-2-(phenyl)quinoli*ne (**4e**). Solid (110 mg, 65%), mp 243–245 °C (ethanol), *R*_F_ 0.15; ν_max_ (neat) 692, 756, 1365, 1612, 3392 cm^−1^; ^1^H-NMR δ_H_ (300 MHz, CDCl_3_) 4.71 (br s, 2H), 7.03 (t, *J* 8.4 Hz, 2H), 7.14–7.21 (m, 5H), 7.27–7.31 (m, 2H), 7.49 (dt, *J* 1.5 and 8.7 Hz, 1H), 7.69 (dt, *J* 1.5 and 8.7 Hz, 1H), 7.78 (dd, *J* 0.6 and 8.7 Hz, 1H), 8.10 (dd, *J* 0.6 and 8.7 Hz, 1H); ^13^C-NMR δ_C_ (75 MHz, CDCl_3_) 114.9, 116.1 (d, ^2^*J*_CF_ 21.3 Hz), 117.4, 120.3, 125.2, 127.4, 127.9, 129.5, 129.6, 130.2, 132.2 (d, ^4^*J*_CF_ 3.7 Hz), 132.8 (d, ^3^*J*_CF_ 8.3 Hz), 141.1, 147.3, 147.6, 158.9, 162.0 (d, ^1^*J*_CF_ 245.6 Hz); *m/z *315 (100, MH^+^), HRMS (ES): MH^+^ found 315.1301. C_21_H_16_FN_2_ requires 315.1293.

#### 3.2.6. 1-Fluoro-4-(4-fluorophenyl)benzene (**2b**) and 4-Amino-2,3-bis(4-fluorophenyl)quinoline (**4f**)

A mixture of **1b** (0.50 g, 1.28 mmol), 4-fluorophenylboronic acid (0.36 g, 2.56 mmol), PdCl_2_(PPh_3_)_2_ (0.04 g, 0.06 mmol), PCy_3_ (0.04 g, 0.13 mmol), 2 M K_2_CO_3_ (2.6 mL) in DMF (6 mL) was treated as described above. Work-up and column chromatography on silica gel (40% ethyl acetate-hexane) afforded **2b** (30 mg, 12%) *R*_F_ 0.90 and **4f** in sequence.

*4-Amino-2,3-bis(4-fluorophenyl)quinoline* (**4f**). Solid (280 mg, 66%), mp 247–249 °C (ethanol), *R*_F_ 0.23; ν_max_ (neat) 616, 797, 881, 1494, 1561, 1616, 3308, 3435 cm^−1^; ^1^H-NMR δ_H_ (300 MHz, CDCl_3_) 4.73 (br s, 2H), 6.88 (t, *J* 8.7 Hz, 2H), 7.05 (t, *J* 8.7 Hz, 2H), 7.16 (t, *J* 8.7 Hz, 2H), 7.27 (t, *J* 8.7 Hz, 2H), 7.49 (dt, *J* 1.2 and 8.4 Hz, 1H), 7.68 (dt, *J* 1.5 and 8.4 Hz, 1H), 7.78 (dd, *J* 1.2 and 8.4 Hz, 1H), 8.07 (d, *J* 8.1 Hz, 1H); ^13^C-NMR δ_C_ (75 MHz, CDCl_3_) 114.6 (d, ^2^*J*_CF_ 21.3 Hz), 114.7, 116.3 (d, ^2^*J*_CF_ 21.4 Hz), 117.3, 120.3, 125.2, 129.7, 130.2, 131.4 (d, ^3^*J*_CF_ 8.0 Hz), 132.2 (d, ^4^*J*_CF_ 3.5 Hz), 132.8 (d, ^3^*J*_CF_ 8.0 Hz), 137.2 (d, ^4^*J*_CF_ 3.5 Hz), 147.4, 147.6, 157.8, 162.1 (d, ^1^*J*_CF_ 246.2 Hz), 162.2 (d, ^1^*J*_CF_ 245.6 Hz); *m/z *(100, MH^+^), HRMS (ES): MH^+^ found 333.1207. C_21_H_15_F_2_N_2_^+^ requires 333.1203.

#### 3.2.7. 1-Fluoro-4-(4-fluorophenyl)benzene (**2b**) and 4-Amino-2-(4-chlorophenyl)-3-(4-fluorophenyl)quinoline (**4g**)

A mixture of **1c** (0.25 g, 0.62 mmol), 4-fluorophenylboronic acid (0.17 g, 1.23 mmol), PdCl_2_(PPh_3_)_2_ (0.02 g, 0.03 mmol), PCy_3_ (0.03 g, 0.06 mmol), 2 M K_2_CO_3_ (1.2 mL) in DMF (8 mL) was treated as described above. Work-up and column chromatography on silica gel (20% ethyl acetate-hexane) afforded **2b** (19.5 mg, 16%), *R*_F_ 0.88 and **4g** in sequence.

*4-Amino-2-(4-chlorophenyl)-3-(4-fluorophenyl)quinoline* (**4g**). Solid (141 mg, 65%), mp 246–248 °C (ethanol), *R*_F_ 0.10; ν_max_ (neat) 762, 833, 1224, 1431, 1490, 1616, 3191, 3310, 3428 cm^−1^; ^1^H-NMR δ_H_ (300 MHz, CDCl_3_) 4.73 (br s, 2H), 7.07 (t, *J* 8.7 Hz, 2H), 7.15–7.20 (m, 3H), 7.24–7.27 (m, 3H), 7.51 (dt, *J* 1.5 and 8.4 Hz, 1H), 7.71 (dt, *J* 1.5 and 8.1 Hz, 1H), 7.79 (dd, *J* 0.6 and 8.4 Hz, 1H), 8.08 (dd, *J* 0.6 and 8.7 Hz, 1H); ^13^C-NMR δ_C_ (75 MHz, CDCl_3_) 114.7, 116.3 (d, ^2^*J*_CF_ 21.3 Hz), 117.4, 120.3, 125.4, 127.9, 129.7, 130.2, 131.0, 132.1 (d, ^4^*J*_CF_ 3.7 Hz), 132.8 (d, ^3^*J*_CF_ 8.0 Hz), 133.6, 139.6, 147.4, 147.6, 157.6, 162.1 (d, ^1^*J*_CF_ 246.5 Hz); *m/z *349 (100, MH^+^), HRMS (ES): MH^+^ found 349.0912. C_21_H_15_FN_2_^35^Cl^+^ requires 349.0908.

#### 3.2.8. 1-Fluoro-4-(4-fluorophenyl)benzene (**2b**) and 4-Amino-3-(4-fluorophenyl)-2-(4-methoxyphenyl)quinoline (**4h**)

A mixture of **1d** (0.20 g, 0.50 mmol), 4-fluorophenylboronic acid (0.14 g, 0.99 mmol), PdCl_2_(PPh_3_)_2_ (0.02 g, 0.02 mmol), PCy_3_ (0.02 g, 0.05 mmol), 2 M K_2_CO_3_ (1.0 mL) in DMF (6 mL) was treated as described above. Work-up and column chromatography on silica gel (20% ethyl acetate-hexane) afforded **2b** (19.6 mg, 21%), *R*_F_ 0.93 and **4h** in sequence.

*4-Amino-3-(4-fluorophenyl)-2-(4-methoxyphenyl)quinoline* (**4h**). Solid (111 mg, 64%), mp 236–240 °C (ethanol), *R*_F_ 0.12; ν_max_ (neat) 609, 764, 834, 1211, 1245, 1591, 1608, 3412, 3477 cm^−1^; ^1^H-NMR δ_H_ (300 MHz, CDCl_3_) 3.75 (s, 3H), 4.73 (br s, 2H), 7.07 (t, *J* 8.7 Hz, 2H), 7.15–7.20 (m, 4H), 7.24–7.27 (m, 2H), 7.51 (t, *J* 8.4 Hz, 1H), 7.71 (t, *J* 8.4 Hz, 1H), 7.79 (dd, *J* 0.6 and 8.7 Hz, 1H), 8.08 (dd, *J* 0.6 and 8.7 Hz, 1H); ^13^C-NMR δ_C_ (75 MHz, CDCl_3_) 55.2, 113.1, 114.8, 116.2 (d, ^2^*J*_CF_ 21.3 Hz), 117.3, 120.3, 124.9, 129.5, 130.1, 131.0, 132.6 (d, ^4^*J*_CF_ 3.2 Hz), 132.8 (d, ^3^*J*_CF_ 8.0 Hz), 133.6, 147.2, 147.6, 158.4, 159.0, 162.0 (d, ^1^*J*_CF_ 245.6 Hz); *m/z *345 (100, MH^+^), HRMS (ES): MH^+^ found 345.1411. C_22_H_18_FN_2_O^+^ requires 345.1403.

#### 3.2.9. 1-Methoxy-4-(4-methoxyphenyl)benzene (**2c**) and 4-Amino-3-(4-methoxyphenyl)-2-phenylquinoline (**4i**)

A mixture of **1a** (0.20 g, 0.54 mmol), 4-methoxyphenylboronic acid (0.16 g, 1.08 mmol), PdCl_2_(PPh_3_)_2_ (0.02 g, 0.03 mmol), PCy_3_ (0.02 g, 0.05 mmol), 2 M K_2_CO_3_ (1.0 mL) in DMF (5 mL) was treated as described above. Work-up and column chromatography on silica gel (20% ethyl acetate-hexane) afforded **2c** and **4i** in sequence.

*1-Methoxy-4-(4-methoxyphenyl)benzene* (**2c**). Solid (30 mg, 16%), mp 179–181 °C (ethanol) (Lit. [35] 178–180 °C), *R*_F_ 0.63.

*4-Amino-3-(4-methoxyphenyl)-2-phenylquinoline* (**4i**). Solid (110 mg, 63%), mp 202–204 °C (ethanol), *R*_F_ 0.08; ν _max_(neat) 704, 765, 1240, 1440, 1510, 1558, 1567, 1607, 3322, 3440 cm^−1^; ^1^H-NMR δ_H_ (300 MHz, CDCl_3_) 3.79 (s, 3H), 4.73 (br s, 2H), 6.87 (d, *J* 8.7 Hz, 2H), 7.11 (d, *J* 8.7 Hz, 2H), 7.16–7.20 (m, 3H), 7.17–7.35 (m, 2H), 7.48 (dt, *J* 0.9 and 7.8 Hz, 1H), 6.68 (t, *J* 0.9 and 8.4 Hz, 1H), 7.78 (dd, *J* 0.6 and 8.7 Hz, 1H), 8.10 (dd, *J* 0.6 and 8.4 Hz, 1H); ^13^C-NMR δ_C_ (75 MHz, CDCl_3_) 55.2, 100.0, 114.4, 115.7, 117.5, 120.3, 125.0, 127.3, 127.6, 128.4, 129.3, 129.7, 130.1, 132.2, 141.4, 147.5, 158.7, 158.9; *m/z *327. (100, MH^+^), HRMS (ES): MH^+^ found 327.1507. C_22_H_19_N_2_O^+^ requires 327.1497.

#### 3.2.10. 1-Methoxy-4-(4-methoxyphenyl)benzene (**2c**) and 4-Amino-2-(4-fluorophenyl)-3-(4-methoxyphenyl)-quinoline (**4j**)

A mixture of **1b** (0.50 g, 1.28 mmol), 4-methoxyphenylboronic acid (0.39 g, 2.56 mmol), PdCl_2_(PPh_3_)_2_ (0.04 g, 0.06 mmol), PCy_3_ (0.04 g, 0.13 mmol), 2 M K_2_CO_3_ (2.6 mL) and DMF (6 mL) was treated as described above. Work-up and column chromatography on silica gel (40% ethyl acetate-hexane) afforded **2c** (50 mg, 18%), *R*_F_ 0.85 and **4j** in sequence.

*4-Amino-2-(4-fluorophenyl)-3-(4-methoxyphenyl)quinoline *(**4j**). Solid (250 mg, 57%), mp 190–192 °C (ethanol), *R*_F_ 0.10; ν_max_ (neat) 611, 763, 799, 1245, 1510, 1558, 1607, 3285, 3427 cm^−1^; ^1^H-NMR δ_H_ (300 MHz, CDCl_3_) 3.81 (s, 3H), 4.74 (br s, 2H), 6.87 (t, *J* 8.7 Hz, 2H), 6.88 (d, *J* 8.7 Hz, 2H), 7.09 (d, *J* 8.7 Hz, 2H), 7.31 (t, *J* 8.7 Hz, 2H), 7.47 (t, *J* 8.4 Hz, 1H), 7.67 (dt, *J* 1.2 and 8.4 Hz, 1H), 7.76 (d, *J* 8.4 Hz, 1H), 8.06 (dd, *J* 0.6 and 8.4 Hz, 1H); ^13^C-NMR δ_C_ (75 MHz, CDCl_3_) 55.2, 114.5 (d, ^2^*J*_CF_ 21.3 Hz), 114.6, 115.5, 117.4, 120.3, 125.0, 128.2, 129.4, 130.1, 131.5 (d, ^3^*J*_CF_ 8.0 Hz), 132.1, 137.5 (d, ^4^*J*_CF_ 3.5 Hz), 147.4, 147.6, 157.9, 158.8, 162.2 (d, ^1^*J*_CF_ 245.3 Hz); *m/z *345 (100, MH^+^), HRMS (ES): MH^+^ found 345.1403. C_22_H_18_FN_2_O^+^ requires 345.1411.

#### 3.2.11. 1-Methoxy-4-(4-methoxyphenyl)benzene (**2c**) and 4-Amino-2-(4-chlorophenyl)-3-(4-methoxyphenyl)-quinoline (**4k**)

A mixture of **1b** (0.30 g, 0.74 mmol), 4-methoxyphenylboronic acid (0.22 g, 1.48 mmol), PdCl_2_(PPh_3_)_2_ (0.03 g, 0.04 mmol), PCy_3_ (0.02 g, 0.07 mmol), 2 M K_2_CO_3_ (1.5 mL) in DMF (6 mL) was treated as described above. Work-up and column chromatography on silica gel (40% ethyl acetate-hexane) afforded **2c** (29.7 mg, 19%), *R*_F_ 0.91 and **4k** in sequence.

*4-Amino-2-(4-chlorophenyl)-3-(4-methoxyphenyl)quinoline* (**4k**). Solid (160 mg, 60%), mp 223–225 °C (ethanol), *R*_F_ 0.26; ν_max_ (neat) 763, 826, 1246, 1511, 1559, 1609, 1633, 3291, 3432 cm^−1^; ^1^H-NMR δ_H_ (300 MHz, CDCl_3_) 3.82 (s, 3H), 4.74 (br s, 2H), 6.89 (d, *J* 8.4 Hz, 2H), 7.09 (d, *J* 8.4 Hz, 2H), 7.16 (d, *J* 8.4 Hz, 2H), 7.27 (d, *J* 8.4 Hz, 2H), 7.48 (t, *J* 7.5 Hz, 1H), 7.67 (t, *J* 7.5 Hz, 1H), 7.77 (d, *J* 8.4 Hz, 1H), 8.06 (d, *J* 8.4 Hz, 1H); ^13^C-NMR δ_C_ (75 MHz, CDCl_3_) 55.2, 114.6, 115.5, 117.5, 120.3, 125.2, 127.8, 120.1, 129.4, 130.1, 131.1, 132.1, 133.4, 139.9, 147.5, 147.6, 157.7, 158.9; *m/z *(100, MH^+^), HRMS (ES): MH^+^ found 361.1111. C_22_H_18_N_2_O^35^Cl^+^ requires 361.1103.

#### 3.2.12. 1-Methoxy-4-(4-methoxyphenyl)benzene (**2c**), 4-Azido-2,3-bis(4-methoxyphenyl)quinoline (**3l**) and 4-Amino-2,3-bis(4-methoxyphenyl)quinoline (**4l**)

A mixture of **1d** (0.20 g, 0.50 mmol), 4-methoxyphenylboronic acid (0.15 g, 0.99 mmol), PdCl_2_(PPh_3_)_2_ (0.02 g, 0.02 mmol), PCy_3_ (0.02 g, 0.05 mmol), 2 M K_2_CO_3_ (1 mL) in DMF (6 mL) was treated as described above. Work-up and column chromatography on silica gel (40% ethyl acetate-hexane) afforded **2c** (30 mg, 28%), *R*_F_ 0.88 and **4l** in sequence.

*4-Azido-2,3-bis(4-methoxyphenyl)quinoline* (**3l**). Solid (16.2 mg, 9%), mp 138–140 °C (ethanol), *R*_F_ 0.46; ν_max_ (neat) 715, 829, 1030, 1175, 1243, 1479, 1607, 2110 cm^−1^; ^1^H-NMR δ_H_ (300 MHz, CDCl_3_) 3.83 (s, 2x3H), 6.88 (d, *J *8.7 Hz, 1H), 6.94 (d, *J *8.7 Hz, 2H), 7.16 (d, *J *8.7 Hz, 1H), 7.22 (d, *J *8.7 Hz, 2H), 7.47 (d, *J* 8.7 Hz, 2H), 7.57 (dt, *J* 1.2 and 8.4 Hz, 1H), 7.75 (dt, *J* 1.2 and 8.4 Hz, 1H), 8.10 (dd, *J* 0.9 and 8.7 Hz, 1H), 8.21 (dd, *J* 0.9 and 8.7 Hz, 1H); ^13^C-NMR δ_C_ (75 MHz, CDCl_3_) 55.2, 55.3, 113.9, 114.1, 123.0, 126.9, 127.7, 128.0, 129.4, 130.3, 131.0, 132.6, 133.4, 138.8, 143.1, 147.7, 158.5, 158.6, 159.7; *m/z *355 (100, MH^+^-N_2_), HRMS (ES): MH^+^-N_2 _found 355.1449. C_23_H_19_N_2_O_2_^+^ requires 355.1447.

*4-Amino-2,3-bis(4-methoxyphenyl)quinoline* (**4l**). Solid (120 mg, 68%), mp 202–204 °C (ethanol), *R*_F_ 0.25; ν_max_ (neat) 758, 1108, 1177, 1241, 1605, 3399, 3488 cm^−1^; ^1^H-NMR δ_H_ (300 MHz, CDCl_3_) 3.75 (s, 3H), 3.81 (s, 3H), 4.70 (br s, 2H), 6.71 (d, *J* 8.7 Hz, 2H), 6.89 (d, *J* 8.7 Hz, 2H), 7.11 (d, *J* 8.7 Hz, 2H), 7.28 (d, *J* 9.0 Hz, 2H), 7.45 (dt, *J* 1.5 and 8.4 Hz, 1H), 7.65 (dt, *J* 1.5 and 8.4 Hz, 1H), 7.75 (d, *J* 8.7 Hz, 1H), 8.07 (dd, *J* 0.6 and 8.4 Hz, 1H); ^13^C-NMR δ_C_ (75 MHz, CDCl_3_) 55.1, 55.2, 113.0, 114.5, 115.6, 117.4, 120.3, 124.8, 128.7, 129.2, 130.0, 130.1, 132.2, 133.9, 147.4, 147.5, 158.5 (2xC), 158.7; MS (EI) *m/z *357 (100, MH^+^); HRMS (ES): MH^+^ found 357.1611. C_23_H_21_N_2_O_2_^+^ requires 357.1603.

### 3.3. Typical Procedure for the Pd(OAc)_2_-Catalyzed Cross-Coupling Reactions of ***1c,d*** with PhB(OH)_2_

#### 3.3.1. Biphenyl (**2a**), 4-Azido-2-(4'-chlorophenyl)-3-phenylquinoline (**3c**) and 4-Amino-2-(4'-chloro-phenyl)quinoline (**4c**)

A mixture of **1c** (0.25 g, 0.62 mmol), phenylboronic acid (0.07 g, 1.23 mmol), Pd(OAc)_2_ (0.01 g, 0.03 mmol) and 2 M K_2_CO_3_ (1.2 mL) in DMF (6 mL) in a two-necked flask equipped with a stirrer bar, rubber septum and a condenser was degassed with argon for 10 min. A balloon filled with argon was connected to the top of the condenser and the mixture was heated at 80–90 °C for 6 h. The mixture was cooled to room temperature and then poured into ice-cold water. The product was taken-up into chloroform and the organic solution was washed with brine, dried (anhydrous MgSO_4_) and then evaporated under reduced pressure. The residue was purified by column chromatography on silca gel (20% ethyl acetate-hexane) to afford three products **2a** (19 mg, 21%), *R*_F_ 0.94; **3c** (29.8 mg, 14%), *R*_F_ 0.50 and **4c** (60 mg, 30%), *R*_F_ 0.14 in sequence.

#### 3.3.2. Biphenyl (**2a**), 4-Azido-2-(4'-methoxyphenyl)-3-phenylquinoline (**3d**) and 4-Amino-2-(4'-methoxyphenyl)quinoline (**4d**)

A mixture of **1d** (0.20 g, 0.50 mmol), phenylboronic acid (0.12 g, 0.10 mmol), Pd(OAc)_2_ (0.01 g, 0.02 mmol) and 2 M K_2_CO_3_ (1.2 mL) in DMF (6 mL) was treated as described above. Workup and column chromatography on silica gel (20% ethyl acetate-hexane, v/v) yielded three products **2a** (20 mg, 26%), *R*_F _0.89; **3d** (30 mg, 14%), *R*_F_ 0.56 and **4d** (80 mg, 50%), *R*_F_ 0.11 in sequence.

## 4. Conclusions

In summary, the direct one-pot palladium-mediated coupling of 2-aryl-4-azido-3-iodoquinolines **1** with arylboronic acids and subsequent reduction of the azido group by the *in situ* generated palladium hydride represents a convenient synthetic strategy for the construction of primary 4-amino-2,3-diaryl-quinolines. The isolation of the symmetrical biaryl derivatives **2** and the observed *in situ* reduction of the azido to amino group using either Pd(OAc)_2_, PdCl_2_(PPh_3_)_2_ or Pd(PPh_3_)_4_ as the Pd(0) catalyst sources provide further support for the involvement of palladium hydride in the reductive elimination step of the catalytic cycle leading to self-coupling of arylboronic acids [32]. At least in our opinion, the results observed in this investigation rule out the possibility of an *in situ *reduction of the azido group via Staudinger reaction with PPh_3_ generated from the catalyst [20] or possible hydrogenation using DMF/water mixture as previously proposed in the literature [29,30]. The versatility of this methodology can be extended to develop a streamlined approach to 2,3-disubstituted primary 4-amino-quinoline libraries and their annulated quinoline derivatives. Moreover, the biaryl scaffold represents a privileged structure for pharmaceutically important compounds [36,37,38].
